# Supplementary material for: Causal Relationship between Aging and Anorexia Nervosa: A White-Matter-Microstructure-Mediated Mendelian Randomization Analysis
Source: Biomedicines. 2024 Aug 16;12(8):1874. doi: 10.3390/biomedicines12081874 (PMC11351342; doi:10.3390/biomedicines12081874)
Supplement: Supplementary file 1 [file biomedicines-12-01874-s001.zip › Supplementary Figure/Figure S8/Leaveoneout plot of MR analysis for TL to ubm-b-1633.pdf]

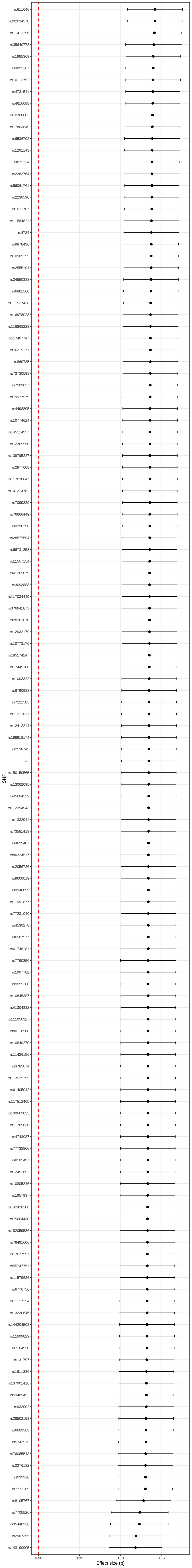
rs611646
rs202034370
rs11412296
rs35640778
rs1985369
rs3891167
rs10112752
rs4731541
rs4616688
rs10768683
rs17803849
rs6536702
rs1291143
rs871134
rs2282764
rs56061761
rs2230590
rs1023767
rs11584821
rs4724
rs9878436
rs10905255
rs2555104
rs34550383
rs6881568
rs111527438
rs16978028
rs116863223
rs117407747
rs76219171
rs869785
rs73730598
rs7209057
rs79977579
rs4498805
rs10774624
rs145114957
rs12369950
rs139795227
rs2977608
rs117630647
rs141214782
rs7099229
rs76666449
rs9398196
rs28577594
rs66731853
rs11557154
rs41269079
rs3093888
rs117034449
rs376641875
rs28363070
rs12932179
rs10773176
rs185174247
rs17445108
rs1003322
rs6790988
rs7221585
rs11212631
rs12412214
rs188918174
rs2538745
All
rs150150565
rs13062095
rs45604339
rs112394943
rs1332941
rs73581419
rs4695407
rs80324517
rs2056726
rs9600019
rs9940099
rs11991877
rs77231040
rs4530278
rs6587577
rs61748181
rs7790856
rs1907702
rs9955360
rs10845387
rs41304832
rs111950327
rs80116508
rs10840270
rs11426156
rs3785074
rs113525195
rs61405042
rs117512405
rs139669835
rs11769630
rs4743037
rs77732866
rs8102497
rs12451892
rs10805346
rs1957937
rs142426306
rs75664430
rs182059586
rs78491606
rs17677991
rs55747751
rs11579626
rs6776756
rs11117354
rs13230646
rs144204502
rs11699829
rs7164950
rs131797
rs1611236
rs137901416
rs59409453
rs932002
rs28502153
rs6669563
rs5742915
rs76065543
rs2276182
rs939916
rs7772289
rs8105767
rs7705526
rs35446936
rs2967355
rs143190905
SNP
0.00
0.05
0.10
0.15
Effect size (b)
